# eEF2K as an important kinase associated with cancer survival and prognosis

**DOI:** 10.1038/s41598-024-78652-4

**Published:** 2024-11-26

**Authors:** Nan Wang, Li-Lan Cen, Zhe Tian, Miao-Miao An, Qian Gu, Xin-Hong Zhou, Yi-He Zhang, Lucas Liu, Jun Zhang, Di Yang, Yong-Zhi Huang, Xi-Dai Long, Qian Yang

**Affiliations:** 1 The Second Surgical Department of Breast Cancer, Tianjin Medical University Cancer Institute & Hospital, National Clinical Research Center for Cancer, Key Laboratory of Cancer Prevention and Therapy, Tianjin, 30071 China; 2 Key laboratory of molecular pathology in Tumors of Guangxi Higher Education Institutions, Department of Pathology, the Affiliated Hospital of Youjiang Medical University for Nationalities, Baise City, 533000 Guangxi Zhuang Autonomous Region China; 3 Youjiang Medical University for Nationalities, Baise City, 533000 Guangxi Zhuang Autonomous Region China; 4 School of Medicine, Nankai University, Tianjin, 300071 China; 5 Atkins Academic & Technology High School, Winston-Salem, NC 27101 USA; 6 Department of Neurosurgery, Peking University International Hospital, Beijing, 102206 China; 7 Present Address: Guangxi Academy of Medical Sciences, Department of Infectious Disease, The People’s Hospital of Guangxi Zhuang Autonomous Region, Nanning, 530021 China; 8 Department of Cancer Biology, Wake Forest University School of Medicine, Atrium Health Wake Forest Baptist Comprehensive Cancer Center, Winston-Salem, NC USA

**Keywords:** eEF2K, Cancer, Phosphorylation, Prognosis, Pan-cancer analysis, Cancer, Computational biology and bioinformatics

## Abstract

Eukaryotic Elongation Factor 2 Kinase (eEF2K), a member of the α-kinase family, services as a crucial negative regulator of protein synthesis, particularly under conditions of cellular stress. A pan-cancer analysis of eEF2K expression, genetic variants, and clinical relevance across multiple tumor types was performed using data from the Cancer Genome Atlas (TCGA) and GEO. Our findings suggest that eEF2K has dual roles in cancer progression, with its expression correlating with patient prognosis. Significant phosphorylation of eEF2 at T57, Y434, and T59 was observed, which may regulate protein synthesis during stress. The elevated T59 phosphorylation in COAD, despite the low eEF2K expression, indicates that this may be regulated by alternative kinases, such as AMPK or mTOR. This suggests that compensatory mechanisms may be involved. In addition to modulating eEF2 phosphorylation, eEF2K is involved in a number of other processes, including peptidyl-serine phosphorylation, the G2/M transition, and the MAPK cascade. The protein products of eEF2K are capable of localizing to the nucleus, cytoplasm, and cytosol, where they bind to a range of proteins, including ATP and calcium ions. These findings provide novel insights into the role of eEF2K in cancer biology and suggest that the targeting of eEF2K and eEF2 phosphorylation may offer promising therapeutic strategies.

**Supplementary Information:**

The online version contains supplementary material available at 10.1038/s41598-024-78652-4.

## Introduction

In recent years, increased numbers of studies have shown that multiple molecular signaling pathways that regulate protein synthesis have a central role in tumor metastasis, progression, and regulation. The eukaryotic elongation factor 2 kinase (eEF2K) molecular signaling pathway is one of the most important signaling pathways. eEF2K is a member of α-kinases^1^ and its activity is dependent on the regulation of calcium/calmodulin (Ca/CaM); hence it is also known as Calcium/Calmodulin-Dependent Protein Kinase III (CaMKIII)^2,3^. In eukaryotes, eEF2 is the only known substrate of eEF2K, which inhibits protein synthesis through phosphorylation of eEF2 on Thr56 and interaction with ribosomes^4^. Growing evidence has shown that eEF2K is activated and overexpressed in many tumors/cancers, promoting cell proliferation, survival, and aggressive tumor characteristics leading to tumor growth and progression^5^. The ability of oncogenes to transform normal cells depends on the reprogramming of many cellular processes, such as cell cycle progression, cell growth, protein synthesis, and metabolism^6^, and eEF2K is also involved in cellular reprogramming processes^5^.

In the present study, we performed a pan-cancer analysis of eEF2K using the TCGA and GEO databases, exploring its potential molecular mechanisms in terms of gene expression, total protein and protein phosphorylation, survival status, genetic alterations, and related pathways of action.

## Results

### eEF2K gene is bidirectionally changed in human tumor

To detect the eEF2K gene expression status in various cancers, we comprehensively screened 7,452 primary tumors and 1,196 normal adjacent-tissue samples spanning 33 cancer or carcinoma types in the TCGA database in the study using the TIMER2 tool (Table 1).

**Table 1:** List of TCGA samples analyzed in this study.

Cancer type	Full name	Tumor samples processed	Normal adjacent samples processed	(P value)	eEF2K phosphorylation site changes	eEF2K expression and tumor survival prognosis
ACC	Adrenocortical carcinoma	79	0	–	–	–
BLCA	Bladder urothelial carcinoma	408	19	2.80E-03	–	DFS (p = 0.0074)
BRCA	Breast invasive carcinoma	1039	112	1.10E-15	S18, S500, S445	–
CESC	Cervical squamous cell carcinoma and endocervical adenocarcinoma	304	3	3.32E-01	–	–
CHOL	Cholangiocarcinoma	36	9	2.26E-09	–	DFS (p = 0.036)
COAD	Colon adenocarcinoma	457	41	5.61E-11	S18, S500, S477, S445, T353	–
DLBC	Lymphoid neoplasm diffuse large B-cell lymphoma	48	0	–	–	–
ESCA	Esophageal carcinoma	184	11	2.68E-01	–	DFS (p = 0.04)
GBM	Glioblastoma multiforme	153	5	2.70E-01	–	–
HNSC	Head and Neck squamous cell carcinoma	520	44	1.38E-03	–	DFS (p = 0.042)
KICH	Kidney Chromophobe	66	25	8.22E-07	–	–
KIRC	Kidney renal clear cell carcinoma	533	72	1.93E-17	S500, S441	OS (p = 0.0071)
KIRP	Kidney renal papillary cell carcinoma	290	32	1.44E-02	–	–
LAML	Acute myeloid leukemia	173	0	–	–	–
LGG	Brain lower grade glioma	516	0	–	–	–
LIHC	Liver hepatocellular carcinoma	371	50	1.18E-13	–	–
LUAD	Lung adenocarcinoma	516	59	1.62E-02	S500, S441	–
LUSC	Lung squamous cell carcinoma	501	51	3.05E-02	–	–
MESO	Mesothelioma	87	0	–	–	–
OV	Ovarian serous cystadenocarcinoma	426	0	–	S462, S464	–
PAAD	Pancreatic adenocarcinoma	178	4	8.89E-01	–	–
PCPG	Pheochromocytoma and Paraganglioma	179	3	6.11E-01	–	–
PRAD	Prostate adenocarcinoma	497	52	3.97E-09	–	–
READ	Rectum adenocarcinoma	166	10	5.71E-03	–	–
SARC	Sarcoma	259	0	–	–	–
SKCM	Skin Cutaneous Melanoma	103	368	6.44E-02	–	–
STAD	Stomach adenocarcinoma	415	35	1.20E-03	–	–
TGCT	Testicular germ cell tumors	150	0	–	–	–
THCA	Thyroid carcinoma	501	59	6.77E-01	–	–
THYM	Thymoma	120	0	–	–	OS (p = 0.017)
UCEC	Uterine Corpus Endometrial Carcinoma	545	35	1.66E-05	S18, S500, S474	DFS (p = 0.019)
UCS	Uterine Carcinosarcoma	57	0	–	–	–
UVM	Uveal Melanoma	80	0	–	–	–

**Fig. 1:**
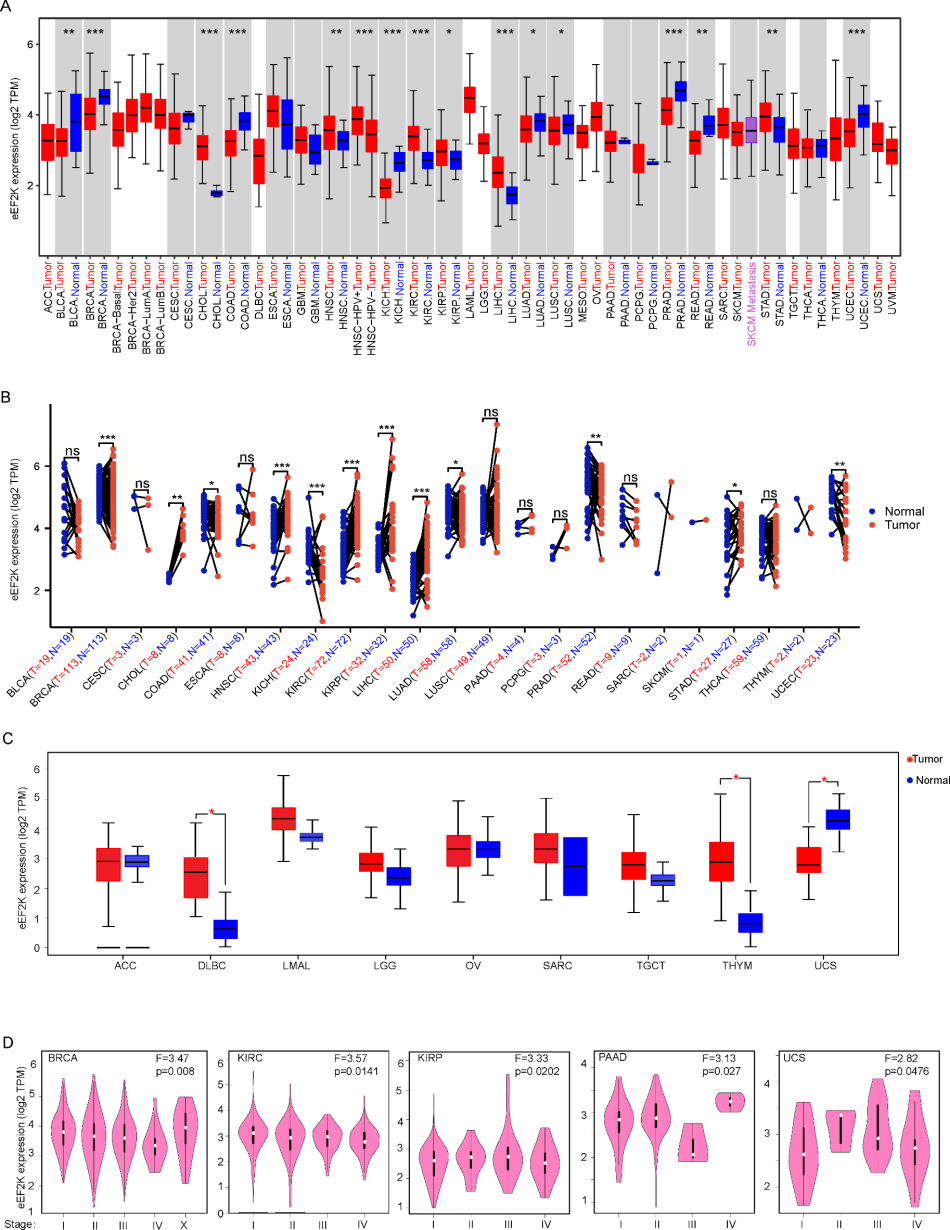
Expression levels of eEF2K gene in different tumors and pathological stages. (**A**) Analysis of eEF2K expression in different tumors or specific tumor subtypes, *p < 0.05; **p < 0.01; ***p < 0.001; ns: not significant. (**B**) Expression levels of eEF2K in 23 paired tumor and normal samples in the TCGA database. (**C**) Box plots of ACC, DLBC, LAML, LGG, OV, SARC, TGCT, THYM, UCS in TCGA prepared by taking corresponding normal tissues in GTEx as control, *p < 0.001. (**D**) Analysis of expression levels of BLCA, KIRC, PAAD, UCS, and KIRP in major pathological stages, using Log2(TPM + 1) as a logarithmic scale.

The expression levels of eEF2K in BRCA, COAD, KICH, PRAD, and UCEC tissues were significantly lower than in normal controls (p < 0.001). This trend is similar in BLCA tissues (p < 0.01) as well as LUAD and LUSC tissues (p < 0.05), but less significantly in the latter two. However, eEF2K expression in CHOL, HNSC, KIRC, and LIHC tissues was notably higher than those in normal controls (p < 0.001), and STAD (p < 0.01) and KIRP (p < 0.05) tissues than their normal controls, but less significantly than tissues such as CHOL (Fig. 1A).

However, the sample sizes of matched adjacent-normal and tumor tissues are not consistent across cancer types in TCGA. To address this, we further analyzed 23 paired tumor specimens and found that the expression of eEF2K mRNA in BRCA, CHOL, HNSC, KICH, KIRC, KIRP, LIHC, and LUAD groups was higher than that in adjacent normal tissues (p < 0.05), while the expression in the COAD, PRAD, STAD, and THMY groups was lower than that in adjacent normal tissues (p < 0.05) (Fig. 1B).

Some cancers have few or no normal tissues. To compensate for the lack of normal samples for some tumor types, TCGA was analyzed jointly with the GTEx database (Fig. 1C). The expression level of eEF2K was higher in DLBC and THYM tissues than in normal controls (p < 0.05). The expression level was lower in UCS tissues than in normal controls (p < 0.05). In ACC, LAML, LGG, OV, SARC, and TGCT groups, there was no significant difference in eEF2K gene expression (p > 0.05).

We also observed that eEF2K gene expression was associated with pathological staging of BRCA, KIRC, KIRP, UCS, and PAAD (p < 0.05), while other cancers were not associated (Fig. 1D).

### Total eEF2K and eEF2 protein bidirectional change and phosphorylation at different sites

**Fig. 2:**
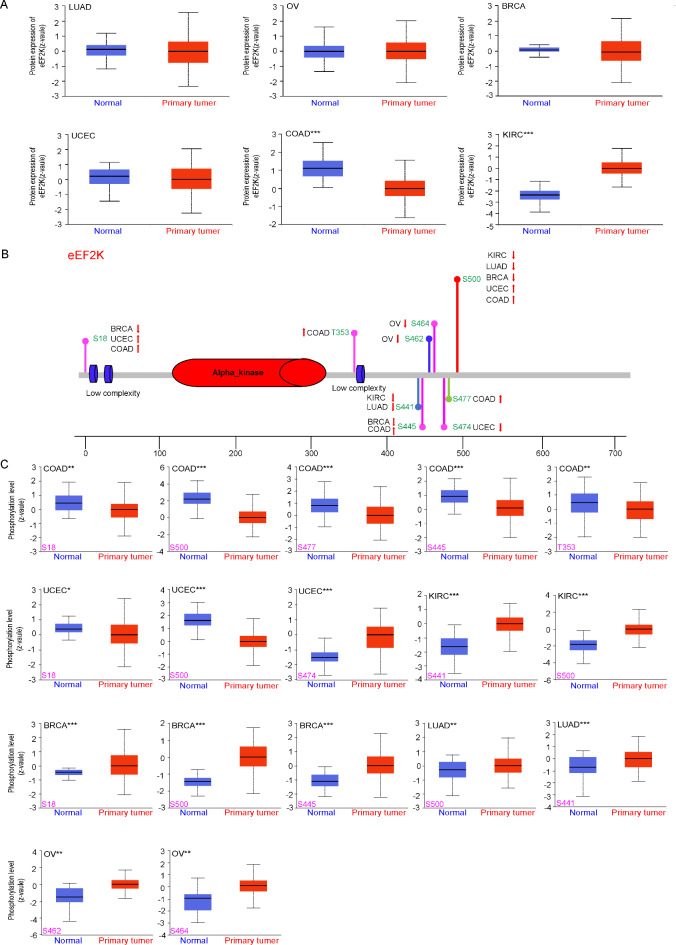
Expression of total eEF2K and phosphorylated proteins in different tumors. (**A**) Analysis of eEF2K total protein expression levels in LUAD, OV, BRCA, UCEC, COAD, KIRC primary cancer tissues, and normal tissues. (**B**) Schematic diagram of eEF2K protein domain showing 6 tumor diseases at each phosphorylation site. (**C**) Expression levels of eEF2K at different phosphorylated protein sites in LUAD, OV, BRCA, UCEC, COAD, KIRC normal tissues, and primary cancer sites.

Next we tested the correlation between eEF2K protein expression and functional change in human cancer. The CPTAC dataset was used to analyze changes in the total protein amount of eEF2K in six kinds of tumors: LUAD, OV, BRCA, UCEC, COAD, and KIRC. Total protein expression of eEF2K was lower than normal in all COAD tissues (p < 0.001), but higher in all KIRC tissues (p < 0.001). The change in the total protein amount of eEF2K in the other four tumors was not enough to be impactful to the study (p > 0.05) (Fig. 2A). To assess the role of eEF2 in various cancer types, we also analyzed the total protein expression levels of eEF2 in several tumors using CPTAC data. eEF2 total protein expression levels were significantly higher than normal tissues in LUAD, OV, BRCA, UCEC, COAD, and KIRC (p < 0.001) (Supplementary Fig. S1A).

Further, utilizing the data in the CPTAC-TCGA datasets containing 364 TCGA cancer samples, we also detected eEF2K and eEF2 protein phosphorylation site in different cancer types (Fig. 2B and Supplementary Fig. S1B) and analyzed the changes of eEF2K and eEF2 protein phosphorylation levels (Fig. 2C, Supplementary Fig. S1C and Table 1). For eEF2K, COAD showed low expression (p < 0.01) at phosphorylation sites S18, S500, S477, S445, T353; UCEC showed low expression (p < 0.05, p < 0.0001) at phosphorylation sites S18, S500 and high expression (p < 0.001) at phosphorylation site S474; BRCA showed high expression ( p < 0.001) at phosphorylation sites S18, S500, S445; OV showed high expression (p < 0.01) at phosphorylation sites S462, S464; KIRC was higher than normal tissues at phosphorylation sites S441 and S500 (p < 0.001); LUAD was higher than normal tissues at phosphorylation sites S441 and S500 (p < 0.001; p < 0.01), suggesting that there is a close association with tumorigenesis at phosphorylation site S500, and it is worth performing further molecular testing to explore the potential role of S500 phosphorylation in tumorigenesis. For eEF2, BRCA showed high protein level (p < 0.01 and p < 0.05) at phosphorylation site T59 and S502; LUAD showed high expression (p < 0.001) at phosphorylation sites T57 and Y434; in KIRC eEF2 protein level was higher than normal tissues at phosphorylation sites T57 and Y434 (p < 0.05 and p < 0.01); in both COAD and OV, eEF2 showed high expression (p < 0.001 and p < 0.05) at phosphorylation site T59.

### The expression of eEF2K in human *cancer* is associated with survival rate

**Fig. 3:**
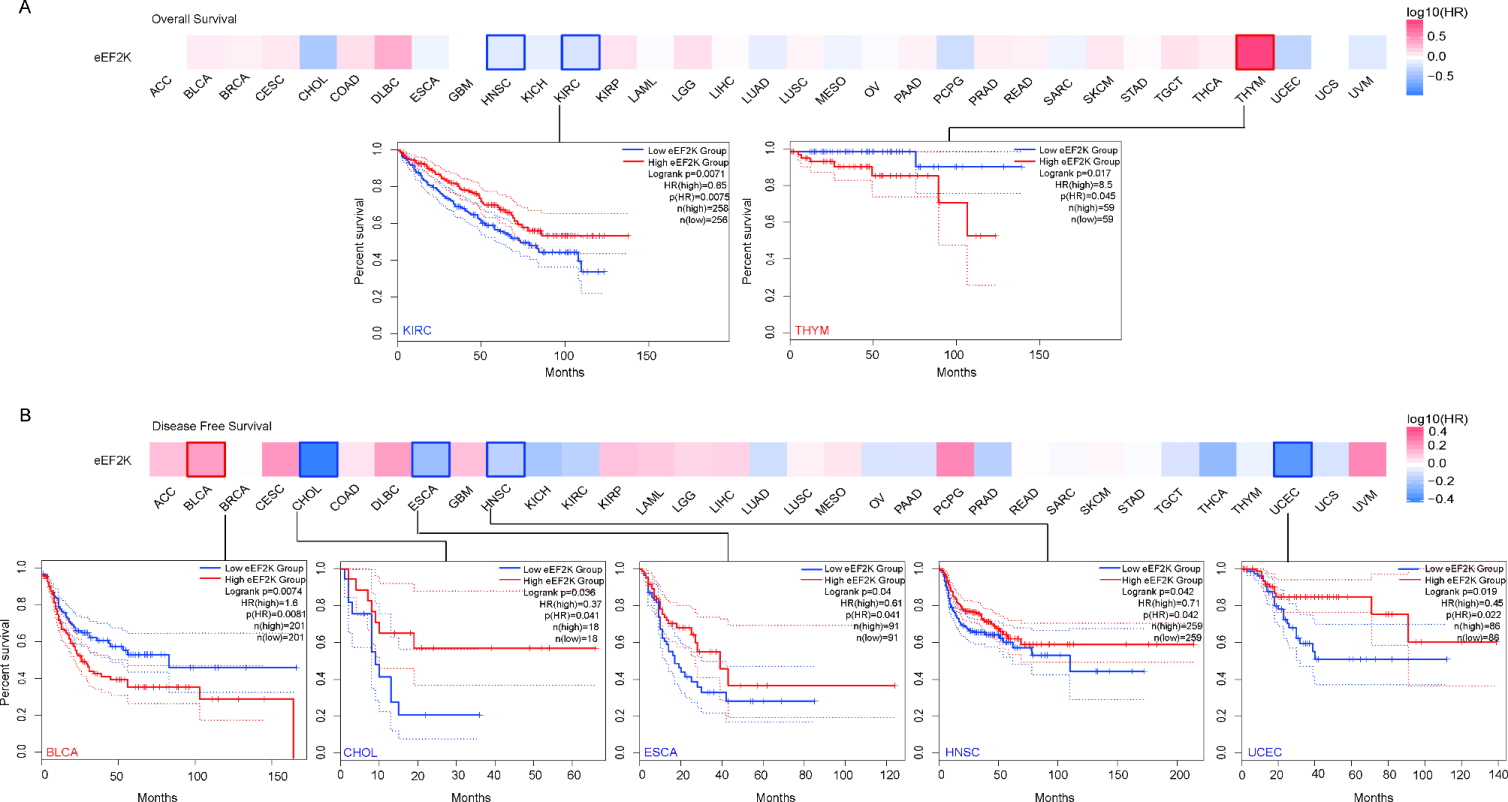
Relationship between eEF2K expression and tumor survival prognosis in TCGA. Kaplan–Meier curves were derived from an analysis of eEF2K gene expression for overall survival (**A**) and disease-free survival (**B**) of different tumors in TCGA.

To analyze the relationship between eEF2K and survival rate in human cancer, tumors were divided into high and low expression groups according to eEF2K expression levels. As shown in Fig. 3A and Table 1, low expression of eEF2K was associated with better overall survival (OS) in THYM (p = 0.017), while high expression of eEF2K was related to better OS in KIRC (p = 0.0071). Likewise, disease-free survival (DFS) analysis data showed (Fig. 3B, Table 1) that low expression of eEF2K was associated with poorer DFS in BLCA (p = 0.0074), and high expression of eEF2K was correlated with poorer DFS prognosis in CHOL (p = 0.036), ESCA (p = 0.04), HNSC (p = 0.042), and UCEC (p = 0.019). The above results suggest that eEF2K differs with the prognosis values in different tumors. Table 2 provides a summary of the eEF2K and eEF2 expression, p-eEF2 site changes and survival outcomes across cancer types.

**Table 2:** Summary of eEF2K and eEF2 expression, phosphorylation, and survival outcomes across cancer types.

Cancer type	eEF2K mRNA expression	eEF2K expression	eEF2 expression	p-eEF2 site changes	eEF2K expression and survival prognosis
BRCA	UP ***	NS	UP ***	T59 UP **, S502 UP *	**OS** NS, **DFS** NS
KIRC	UP ***	UP ***	UP ***	T57 UP *, Y434 UP **	***OS*** **, ***DFS*** NS
LUAD	UP *	NS	UP ***	T57 UP ***, Y434 UP ***	***OS*** NS, ***DFS*** NS
COAD	DOWN *	DOWN ***	UP ***	T59 UP ***	**OS** NS, **DFS** NS

Bold & Italic: Low eEF2K group is worse, Bold: High eEF2K group is worse. Statistical significance is indicated: NS no significance; *p < 0.05; **p < 0.01; ***p < 0.001.

### Genetic variation in human tumor

**Fig. 4:**
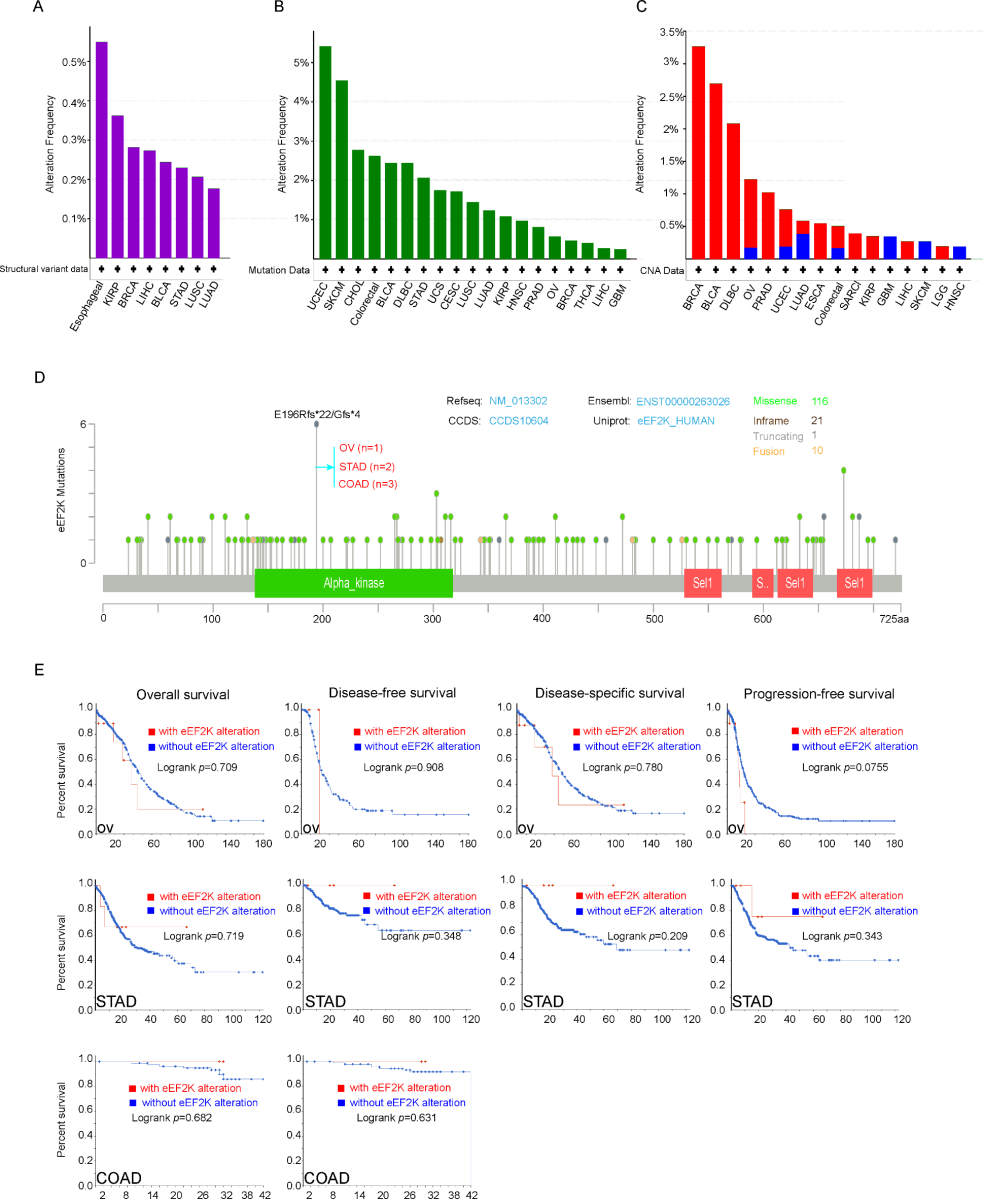
eEF2K variant types of different tumors in TCGA. (**A**) Shows the structural variant type. (**B**) Shows the type of gene mutation (Mutation). (**C**) Copy number (CNA) type shown. (**D**) Shows the rate of change at the mutation site and the mutation site with the highest frequency of eEF2K alteration (E196Rfs*22/Gfs*4). (**E**) Potential correlation between this mutation site and OS, PFS, DFS, DSS of OV, STAD, and COAD.

Mutations occur in the eEF2K genes in the cancer tumor samples. In the TCGA cohort, esophageal cancer patients have the highest frequency of structural variable (Fig. 4A), while the highest frequency of SNV mutation occurs in the UCEC patients. (>6%), followed by SKCM (>5%) and BRCA (Fig. 4B). Fig. 4C shows that the cancer type with the highest “copy number (CNA)” in eEF2K is BRCA and BLCA. Fig. 4D further shows the types and sites of eEF2K genetic variants. E196Rfs*22/Gfs*4 in the structural domain of the Alpha kinase family (Alpha kinase) was detected in one OV, two STAD, and three COAD cases capable of causing a splicing mutation at site 196 of the eEF2K gene.

We explored the potential association between eEF2K gene alterations and clinical prognosis of patients with different types of cancer. Compared with cases without eEF2K gene alterations, eEF2K changes are not significantly associated with OS (p = 0.709), DFS (p = 0.908), DSS (p = 0.780), and PFS (p = 0.0755) in patients with OV; OS (P = 0.719), DFS (p = 0.348), DSS (p = 0.209), and PFS (p = 0.343) in patients with STAD; and OS (p = 0.709) and DFS (p = 0.709) in patients with COAD (Fig. 4E).

### Enrichment analysis of eEF2K-related genes

**Fig. 5:**
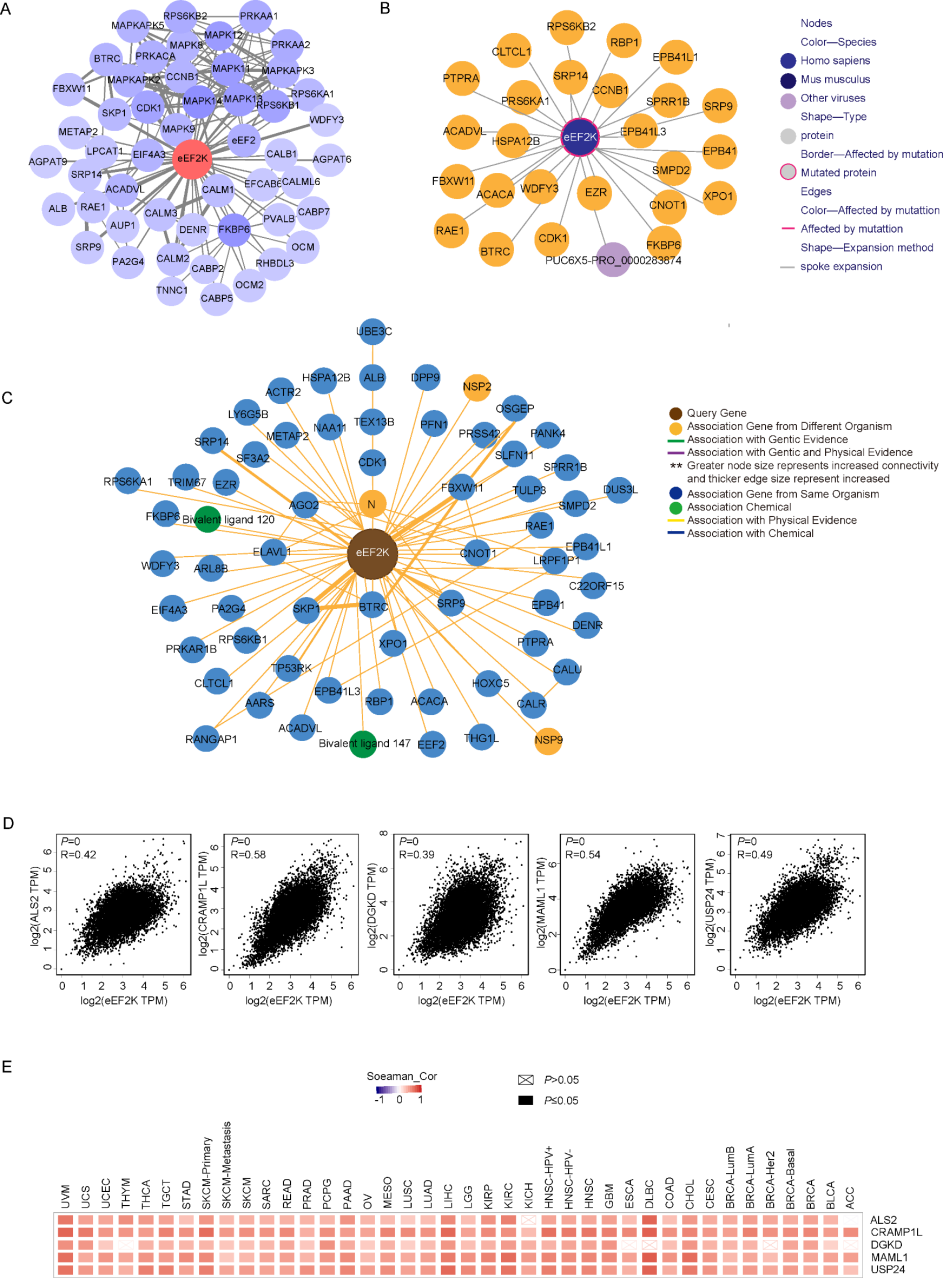
eEF2K binding proteins identified from existing experiments were obtained using the STRING, IntAct and BioGRID tool. (**A**) Protein–protein interaction network of eEF2K, identified using the STRING tool. (**B**) Protein–protein interactions of eEF2K identified using the IntAct database. (**C**) Protein–protein interactions of eEF2K identified using the BioGRID database. (**D**) Correlation analysis between eEF2K and the top 100 correlated genes identified via GEPIA2 and TCGA databases. (**E**) Heatmap showing positive associations of eEF2K with these genes across various cancer types.

We further investigated the molecular mechanism of the eEF2K gene in tumorigenesis. Utilizing the STRING tool, a total of 50 proteins with an experimental validation of binding to eEF2K were screened. The interaction network of these proteins is shown in Fig. 5A. Protein-protein interaction (PPI) analysis of eEF2K was also conducted using the InTAct and BioGRID databases. The interaction networks of these proteins are shown in Fig. 5B,C. We found that CDK1, eEF2, RAE1, SRP9, SRP4, FBXW11, WDFY3, ACADVL, FKBP6, CCNB1, and BTRC were consistently interacting with eEF2K across all three databases. Correlation analysis was performed using GEPIA2 and the TCGA database and the top 100 genes correlated with eEF2K were selected. The expression levels of eEF2K were positively correlated with those of ALS2 (R=0.42), CRAMP1L (R = 0.58), DGKD (R = 0.39), MAML1 (R = 0.49), and USP24 (R = 0.54) (Fig. 5D). The heat map shows (Fig. 5E) that eEF2K is positively associated with the aforementioned genes in the majority of cancer types (Table 3).

**Table 3:** eEF2k correlation with top 5 genes in each cancer information.

Abbreviation	Tumor samples processed	ALS2 (P value)	CRAMP1L (P value)	DGKD (P value)	MAML1 (P value)	USP24 (P value)
ACC	79	0.822739	3.10E-08	0.34801	7.42E-06	0.005638
BLCA	408	4.04E-15	1.30E-26	4.46E-06	1.58E-22	9.68E-22
BRCA	1100	2.85E-58	4.63E-109	2.07E-59	2.01E-53	7.09E-49
BRCA-Basal	191	3.95E-09	5.62E-18	1.24E-10	4.30E-11	3.66E-13
BRCA-Her2	82	3.63E-05	8.29E-08	0.051986	0.00250	4.25E-05
BRCA-LumA	568	4.99E-33	5.65E-64	2.75E-20	3.80E-27	6.06E-37
BRCA-LumB	219	1.51E-10	3.91E-14	3.01E-08	1.37E-07	1.46E-10
CESC	306	5.02E-14	1.06E-22	8.59E-12	1.50E-22	8.40E-24
CHOL	36	0.001460	8.31E-05	0.001015	6.08E-07	1.10E-05
COAD	458	2.58E-12	1.62E-37	2.16E-12	2.00E-35	8.29E-20
DLBC	48	3.74E-10	6.96E-11	0.077642	6.84E-12	1.22E-10
ESCA	185	5.24E-06	2.43E-19	0.093616	2.38E-11	3.30E-08
GBM	153	4.53E-10	1.23E-18	2.59E-12	1.71E-15	1.87E-11
HNSC	522	4.77E-26	4.97E-72	6.02E-28	1.66E-52	3.31E-54
HNSC-HPV-	422	4.42E-22	4.54E-54	1.54E-22	1.41E-37	3.96E-40
HNSC-HPV +	98	1.95E-06	3.29E-16	9.12E-05	2.51E-11	8.60E-13
KICH	66	0.059474	0.0063265	0.001153	1.11E-05	0.00015
KIRC	533	1.41E-60	3.50E-45	8.85E-35	7.29E-89	1.22E-78
KIRP	290	7.50E-24	1.60E-28	6.64E-12	4.80E-32	2.80E-29
LGG	516	2.46E-14	8.88E-29	9.08E-14	5.32E-41	1.81E-50
LIHC	317	4.76E-36	2.69E-67	7.11E-28	1.50E-46	1.45E-65
LUAD	515	2.78E-14	3.01E-41	6.27E-15	6.74E-30	2.50E-35
LUSC	508	1.79E-09	2.30E-47	3.52E-08	2.82E-23	5.34E-28
MESO	87	0.00134	1.84E-10	0.000248	1.69E-06	8.96E-09
OV	303	5.72E-11	2.75E-17	4.31E-06	2.72E-09	3.75E-19
PAAD	179	6.56E-15	1.41E-12	9.15E-09	5.39E-17	2.03E-19
PCPG	181	3.76E-07	1.18E-16	1.81E-15	1.42E-13	3.72E-14
PRAD	498	2.85E-18	2.24E-20	2.42E-10	1.49E-17	6.05E-42
READ	166	2.66E-07	8.31E-17	5.73E-09	7.23E-11	2.66E-10
SARC	260	2.72E-10	2.06E-21	1.91E-09	8.75E-14	3.59E-15
SKCM	471	3.17E-19	1.05E-40	1.88E-12	2.15E-15	6.21E-31
SKCM-Metastasis	368	6.75E-12	3.40E-26	3.03E-08	1.19E-07	6.79E-20
SKCM-Primary	103	1.12E-08	7.97E-18	0.000125	5.98E-11	2.40E-13
STAD	415	1.17E-10	2.40E-34	1.97E-14	1.31E-37	7.07E-24
TGCT	150	1.86E-09	1.81E-17	1.91E-06	1.52E-10	2.99E-18
THCA	509	3.72E-35	1.69E-33	2.66E-19	6.17E-39	3.18E-59
THYM	120	1.63E-11	6.13E-10	0.41178	4.46E-07	7.49E-06
UCEC	545	4.08E-28	1.45E-39	4.50E-10	5.45E-40	1.21E-33
UCS	57	0.000230	2.33E-08	3.39E-05	0.00111	4.19E-05
UVM	80	8.86E-12	2.43E-16	8.47E-08	9.11E-14	7.18E-13

**Fig. 6:**
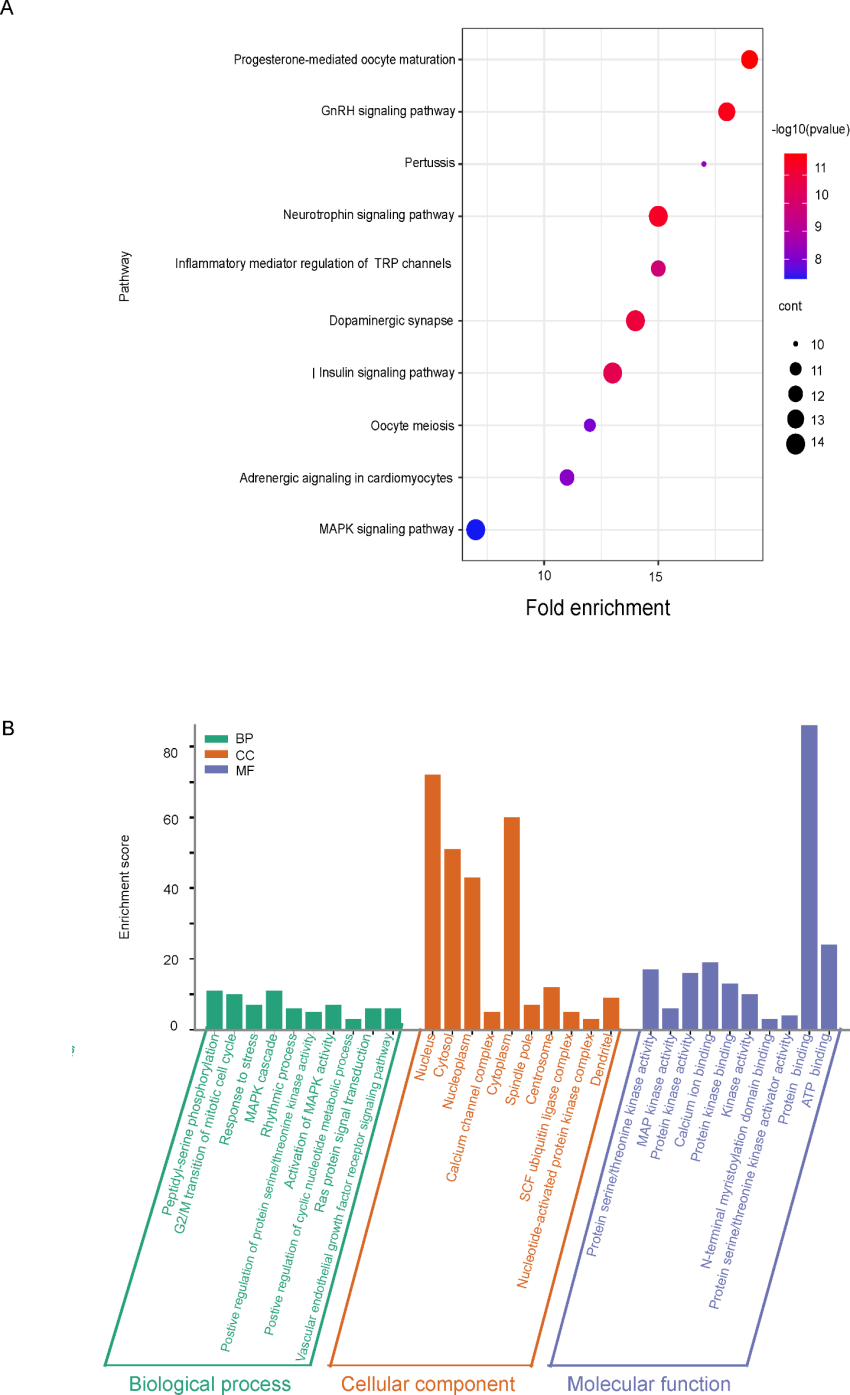
eEF2K KEGG enrichment analysis and GO analysis. KEGG pathway analysis^28–30^ (**A**) and GO analysis (**B**) were performed according to the eEF2K binding and interacting genes.

The above two sets of data were combined for KEGG and GO enrichment analysis. The KEGG data suggests that the role of eEF2K in cancer pathogenesis may be related to the GnRH, neurotrophin, insulin, and MAPK signaling pathways (Fig. 6A). GO enrichment analysis further showed that eEF2K is involved in the biological processes of peptidyl-serine phosphorylation, G2/M transition of the mitotic cell cycle, MAPK cascade, and most of the gene encoding products are localized in the nucleus, cytoplasm, and cytosol, and perform molecular functions to bind proteins, ATP and calcium ions (Fig. 6B).

## Discussion

In a literature search, we failed to retrieve any publication on pan-cancer analysis of eEF2K from the perspective of the tumor as a whole. Therefore, we performed a more comprehensive and integrated detection analysis of the eEF2K gene in different tumors based on data from TCGA, CPTAC, and GEO databases, as well as molecular characterization of gene expression, protein phosphorylation, and genetic alterations. We found both high and low expression of eEF2K in cancer tissues and normal tissues through the TCGA database, however, some cancers in the database lacked adjacent normal or paracancerous tissues for control including, but not limited to, ACC and DLBC, so TCGA was analyzed jointly with the GTEx database.

We have found that the eEF2K expression in tumors plays a dual role, which can not only contribute to the growth of tumors in vivo but also inhibit tumorigenesis. EEF2K gene expression was higher in BRCA, LUAD, CHOL, HNSC, KIRC, KIRP, and LIHC cancer tissues than normal tissues and lower in BLCA, COAD, KICH, LUSC, PRAD, STAD, and UCEC cancer tissues. Recent studies are consistent with our findings that eEF2K is highly expressed in a variety of solid tumors, including breast, pancreatic, lung, and hepatocellular carcinomas^7–10^, and may play a tumor-suppressive role in colorectal, endometrial, and gastric cancer tissues^11–13^. Notably, the eEF2K phosphorylation site S474 reported in our study was highly expressed in endometrial cancer, so it can be assumed that different phosphorylation sites perform different functions. However, its role in most types of cancer has not been determined. Interestingly, in TCGA, eEF2K mRNA expression showed no difference between PAAD patients and normal tissues, but eEF2K mRNA was associated with the pathological staging of PAAD, which may be related to the fact that there were no more than 200 cases of PAAD in TCGA, with only 4 cases in normal tissues. and no PAAD-related proteins were expressed in the CPTAC database. Perhaps a larger sample size or other tests may be needed to verify the above findings. Previous studies have found high expression of eEF2K in glioblastoma multiforme^14^ and brain glioma^15^. This difference may be caused by the low number of GBM patient cases in TCGA. Previous studies have shown that the physiological roles of eEF2K vary significantly across different cancer types, suggesting a context-dependent function. This may be related to its role in regulating protein synthesis and other cellular processes, but is also influenced by several factors, including the specific cancer cell type, the tumor microenvironment, and the activation level of eEF2K. For example, in breast cancer (BRCA), especially triple‐negative breast cancer (TNBC), eEF2K is often overexpressed, contributing to poor patient prognosis by promoting cancer cell survival under conditions of metabolic stress. This overexpression can also promote cell migration and invasion, thus playing a critical role in tumor progression^16,17^. The same observation has been achieved in lung adenocarcinoma (LUAD) where eEF2K is typically overexpressed to promotes the migration, invasion, and angiogenesis of LUAD cells, thereby driving tumor progression^18^. In contrast, in colorectal cancer (CRC), eEF2K has been shown to act as a tumor suppressor by inhibiting cancer cell growth through autophagy^11^. Regardless of whether eEF2K is up-regulated or down-regulated in tumor tissues, it is expected to be a biomarker for poor tumor prognosis and a potential molecular target for targeted therapy.

We first explored the molecular mechanisms of eEF2K protein in LUAD, OV, BRCA, UCEC, COAD, and KIRC from the perspective of total and phosphorylated proteins using the CPTAC database, finding that they play a regulatory role both in COAD and KIRC, while it is a phosphorylated protein that performs the function for the rest of the tumors. For COAD, we analyzed the TCGA-COAD (t=457) and TCGA-COAD (n=41) datasets and found that eEF2K was expressed in very small amounts in COAD tissues for mRNA, total protein, and multiple phosphorylation sites (S18, S500, S477, S445, T353). While other diseases such as BRCA, UCEC, KIRC, and LUAD showed differences at site S500, we cannot exclude a potential role of eEF2K in said phosphorylation site for each TCGA tumorigenesis and needs to be further explored to obtain more evidence. The low expression of eEF2K in COAD suggests that enhanced eEF2K activity is beneficial for colorectal cancer patients, which is consistent with previous findings^19^. A similar study was done by Tung H. Ng et al., where they used quantitative RT-PCR and Western blots to detect the expression of eEF2K in primary tumors and paracancerous tissues of 20 patients with colorectal cancer and found that mRNA and protein levels of eEF2K were significantly down-regulated in colorectal cancer tissues. EEF2K has tumor-suppressing effects upon colorectal cancer, in which silencing of eEF2K induces survival-promoting autophagic responses through the AMPK-ULK pathway and promotes the growth of colorectal cancer by increasing cell size, survival rate, and clonogenicity^11,20^. On the contrary, overexpression of eEF2K reduced the viability of colon cancer cells and enhanced the antitumor effect of the chemotherapy drug Oxaliplatin^20^. In vivo studies conducted by Faller and his colleagues also showed that eEF2K activation led to growth arrest in APC-deficient colorectal adenomas^19^. However, in TCGA, we failed to detect a significant correlation between eEF2K expression and clinical prognosis in terms of pathological stage, OS, and DFS of colorectal cancer patients. In the Alpha-kinase domain, COAD had the highest rate of mutations in E196Rfs*22/Gfs*4, but we also did not find an effect of eEF2K mutation status on OS (P=0.682) and DFS (P=0.631) in COAD patients. In contrast, in other diseases of ovarian cancer and gastric cancer, eEF2K mutation status was not significantly correlated with the clinical prognosis of OS, PFS, RFS, and DSS.

Some studies have reported the role of high eEF2K expression in carcinogenesis, metastasis, and invasion of breast cancer^21,22^. Interestingly, based on data from the TCGA database, we found that eEF2K gene expression and protein expression in invasive breast carcinoma were contradictory. Such discrepancy is caused by multiple reasons. a protein is the end product of the gene and the true executor of gene function as well, but mRNA is unreliable when reflecting the quality and quantity of protein due to its isolated storage, transport, degradation, translation regulation, and post-translational processing of the product. The only obvious line between the DNA and the final protein is the nascent peptide chain, but that is not the final protein. After the synthesis of the nascent peptide chain, there are various processing and modification processes including phosphorylation and glycosylation that blur the line between gene and protein. The sheer processing of a large number of proteins can not only change their structure but also serve as the structural basis for the implementation of their function and regulation, which cannot be predicted from the genetic level but can only be analyzed through the final functional protein. In conclusion, it is normal for gene and protein expression levels to be inconsistent, and the protein expression level is more persuasive.

In addition to the regulatory role of eEF2K, our analysis revealed significant alterations in the total protein expression and phosphorylation of eEF2 across several cancer types. Specifically, eEF2 total protein expression was consistently elevated in tumors such as BRCA, KIRC, LUAD, and COAD (Table 2). This suggests that eEF2 plays a crucial role in promoting protein synthesis and sustaining rapid cancer cell proliferation in these tumors. Phosphorylation of eEF2, particularly at Thr56, has long been recognized as a critical modulator of protein elongation during translation. Phosphorylation at Thr56 prevents eEF2 from binding to ribosomes, thereby inhibiting ribosomal translocation and halting protein elongation. This mechanism is particularly crucial under stress conditions such as energy deprivation, hypoxia, or nutrient scarcity, conditions frequently encountered in tumor microenvironments^3,23^. Beyond Thr56, other phosphorylation sites’ roles are less clearly defined in the context of cancer. Our data indicate elevated phosphorylation at sites such as T57 and Y434 in cancers like KIRC and LUAD, may contribute to the regulation of protein synthesis under stress conditions. Like Thr56, T57 phosphorylation inhibits protein elongation, representing an adaptive mechanism that allows cancer cells to conserve energy during nutrient deprivation, hypoxia, or other stress-related conditions. Interestingly, in COAD, despite low eEF2K expression, T59 phosphorylation of eEF2 remains elevated, suggesting the involvement of alternative kinases or compensatory pathways. AMPK or mTOR may phosphorylate eEF2 in the absence of active eEF2K, ensuring that protein synthesis is finely modulated to promote tumor survival under adverse conditions. Targeting eEF2 phosphorylation directly, particularly at T56, T57, or T59, could be a promising therapeutic strategy, potentially impairing cancer cell survival and inhibiting tumor progression.

We performed a protein-protein interaction (PPI) analysis for eEF2K using the STRING, IntAct, and BioGRID databases. Our results revealed consistent interactions with proteins such as CDK1, eEF2, RAE1, SRP9, SRP4, FBXW11, WDFY3, ACADVL, FKBP6, CCNB1, and BTRC across all three databases. It is worth noting that PPI networks generated by different tools (e.g., STRING, IntAct, and BioGRID) may vary, due to possible factors such as differences in data sources, variations in the definition of protein-protein interactions, distinct algorithms and prediction models, updates and time of database curation, and thresholds and filtering criteria used to display interactions.

In this study, we investigated the expression of eEF2K in different tumor types and its potential role in cancer progression. We also utilized the Human Protein Atlas (HPA) to confirm eEF2K RNA and protein expression in various cancer tissues. We observed that (1) in the HPA COAD cohort, lower eEF2K expression is associated with improved survival outcomes, consistent with our observations from the TCGA cohort; (2) in the HPA BRCA cohort, we observed high eEF2K mRNA expression levels in the tumor samples, consistent with the TCGA BRCA cohort. However, the protein level presented in this cohort is mostly low, probably due to a combination of differences in sample size and cohort, data source and methodologies and statistical power; (3) in the Lung cancer (LUAD and LUSC) cohort, the observations regarding mRNA and protein levels as well as its prognostic roles closely align with those from the TCGA cohort.; and (4) in the KIRC cohort, we consistently observed that patients with high eEF2K expression have better survival outcomes, although the changes of eEF2K mRNA expression in this cohort is moderate and protein expression is lower, compared to the normal samples. All of these highlight the prognostic significance of eEF2K and supporting its potential utility as a therapeutic target in various cancer types.

eEF2K has been found to be related with various malignancies, including triple-negative breast cancer, glioma, pancreatic cancer, lung cancer and neuroblastoma^24–26^. Studies indicate that eEF2K is associated with key oncogenic processes, such as tumor proliferation, survival, tumorigenesis, invasion and drug resistance^21,27^. For example, microRNA 603 inhibits tumor formation in triple-negative breast cancer through the targeted suppression of eEF2K^16^. Additionally, eEF2K has been reported to enhance the proliferation of ovarian cancer cells, with its elevated expression correlating with poor clinical outcomes^27^. In hepatocellular carcinoma, eEF2K promotes angiogenesis via the PI3K/Akt and STAT3 signaling pathways^10^. Similarly, a positive correlation has been observed between eEF2K expression and proliferation, invasion and metastasis in lung cancer^8^. These findings highlight the critical role of eEF2K in cancer biology and underscore its potential as a therapeutic target in cancer treatment.

At the present time, the exact role of eEF2K in carcinogenesis, the underlying mechanisms involved, and whether it can play a role in the pathogenesis of different tumors through some common molecular mechanisms are still unclear. For the first time, we investigated the pan-cancer analysis of eEF2K and eEF2, statistically correlating its expression was with pathological staging, clinical prognosis, and protein phosphorylation, which helps understand the role of eEF2K in tumorigenesis from the perspective of clinical tumor samples. However, the association with genetic alterations needs to be further demonstrated.

## Methods

To explore the differences in eEF2K gene expression among different cancers (or specific cancer subtypes) and normal tissues, the expression profiles of TCGA tumors were tapped through the Tumor Immune Estimation Resource, Version 2 (TIMER2) network (http://timer.cistrome.org). For certain tumors with no normal or highly restricted normal tissue, such as TCGA-AML (Acute Myeloid Leukemia), TCGA-LGG (Brain Lower Grade Glioma), etc. The Genotype-Tissue Expression Database (GTEx database) of the Gene Expression Profiling Interactive Analysis, version 2 (GEPIA2) web server (http://gepia2.cancer-pku.cn/#index) was used to obtain a box plot of gene expression differences, with the parameters set as: the Log2FC cutoff =1 and the P-value cutoff =0.01. In addition, a violin curve of eEF2K gene expression in different pathological stages of TCGA tumors was obtained according to the "pathological staging diagram" of GEPIA2. The gene expression levels of eEF2K were displayed using the Log2 (TPM+1) scale and plotted as a violin plot.

The UALCAN database (http://ualcan.path.uab.edu/analysis-prot.html) was used to analyze the expression of total and phosphorylated proteins of eEF2K and eEF2 between primary tumors and normal tissues via the CPTAC (Clinical Proteomic Tumor Analysis Consortium) dataset. In this study, the valid data sets were selected from six types of tumors, including breast cancer (BRCA), colon cancer (COAD), Kidney renal clear cell carcinoma (KIRC), and the like. The ID corresponding to the eEF2K and eEF2 protein was found via the Universal Protein (UniProt) software (https://www.uniprot.org/), which was entered into the SMART tool (http://smart.embl.de/) to find the protein domain.

Significant difference data in eEF2K overall survival (OS) and disease-free survival (DFS) were obtained among all TGCA cancer types using the GEPIA2 “survival graph” module. Appropriate expression thresholds were chosen to distinguish between high and low expression populations (High Cutoff=50%; Low Cutoff=50%). Hypothesis testing was performed using the Mentel-Cox test, and survival curves (Kaplan-Meier curves) were obtained using the “survival analysis” module of GEPIA2.

The cBioportal software was used to access the biology website (https://www.cbioportal.org/). The TCGA Pan-cancer Atlas was applied to a total of 10,967 samples from over 28 types of diseases to investigate the characteristics of eEF2K gene changes and to observe the results, which were mutation type, alteration frequency, and copy number alteration (CNA) of eEF2K in all TCGA tumors, while also comparing data on the differential effects of genetic alterations in eEF2K on OS, DFS, progression-free survival (PFS), and disease specific survival.

We used STRING (https://string-db.org) to search eEF2K’s name and organism (Homo sapiens), with the following parameters set: minimum required interaction score set to a low confidence level of 0.150; line color indicates the type of interaction evidence; maximum number of interaction objects displayed: no more than 50; source of interaction: experiments. Finally, we selected 50 experimentally validated eEF2K binding proteins.

Correlation analysis was performed using GEPIA2, the top 100 genes correlated with eEF2K were selected based on TCGA and GTEx datasets, and from which the related genes were selected for Pearson correlation analysis of paired genes with eEF2K. Log2TPM was adopted for the scatter plot, giving P value and correlation coefficients R. Correlation heat maps between the selected genes and eEF2K were made based on the associated genes selected in the previous step.

Protein-protein interaction (PPI) analysis for eEF2K was replicated using the IntAct database (http://www.ebi.ac.uk/intact/). The query was performed by inputting “eEF2K” as the target gene name, specifying the organism as Homo sapiens. The following parameters were set: the minimum required interaction score was adjusted to a low confidence level (0.150), and to control the scope and readability of the results, the maximum number of displayed interaction partners was limited to 50. This resulted in the identification of 26 experimentally validated proteins that interact with eEF2K.

Further PPI analysis was conducted using the BioGRID database (https://thebiogrid.org/). The same query parameters were applied, specifying “eEF2K” and “Homo sapiens”. For BioGRID, the minimum interaction strength was set to 1, and the maximum number of displayed interaction partners was capped at 50. This search yielded 63 experimentally verified proteins interacting with eEF2K.

In addition, two sets of data were combined for pathway analysis of KEGG (Kyoto Encyclopedia of Genes and Genomes)^28–30^. The data of functional annotation maps were obtained by uploading the gene list to DAVID (Database for Annotation, Visualization, and Integrated Discovery) and selecting "OFFICIAL_GENE_SYMBOL " and “Homo sapiens” as gene identifiers and species respectively. Finally, the bubble diagram showing the enrichment pathways was drawn using an online platform for data analysis and visualization (http://www.bioinformatics.com.cn). The platform was also used to perform Gene Ontology (GO) Enrichment Analysis and visualize the data of Biological process (BP), Cellular component (CC), and Molecular function (MF).

## Electronic supplementary material

Below is the link to the electronic supplementary material.

Supplementary Material 1

## Data Availability

The datasets analyzed during the current study are available in the GEPIA database (http://gepia2.cancer-pku.cn/#index), STRING database (https://string-db.org/), IntAct database (http://www.ebi.ac.uk/intact/), BioGRID database (https://thebiogrid.org/), cBioPortal database (http://www.cbioportal.org), UALCAN database (http://ualcan.path.uab.edu/analysis-prot.html), TCGA database (https://tega-data.nci.nih.gov/tega), or TIMER database (http://timer.cistrome.org/).
