# Supplementary material for: eEF2K as an important kinase associated with cancer survival and prognosis
Source: Sci Rep. 2024 Nov 26;14:29284. doi: 10.1038/s41598-024-78652-4 (PMC11599947; doi:10.1038/s41598-024-78652-4)
Supplement: Supplementary file 1 — Supplementary Material 1 [file 41598_2024_78652_MOESM1_ESM.pdf]

Supplementary Information

This supplementary information contains the following contents:

Supplemental Figures

Supplemental Figure Legends

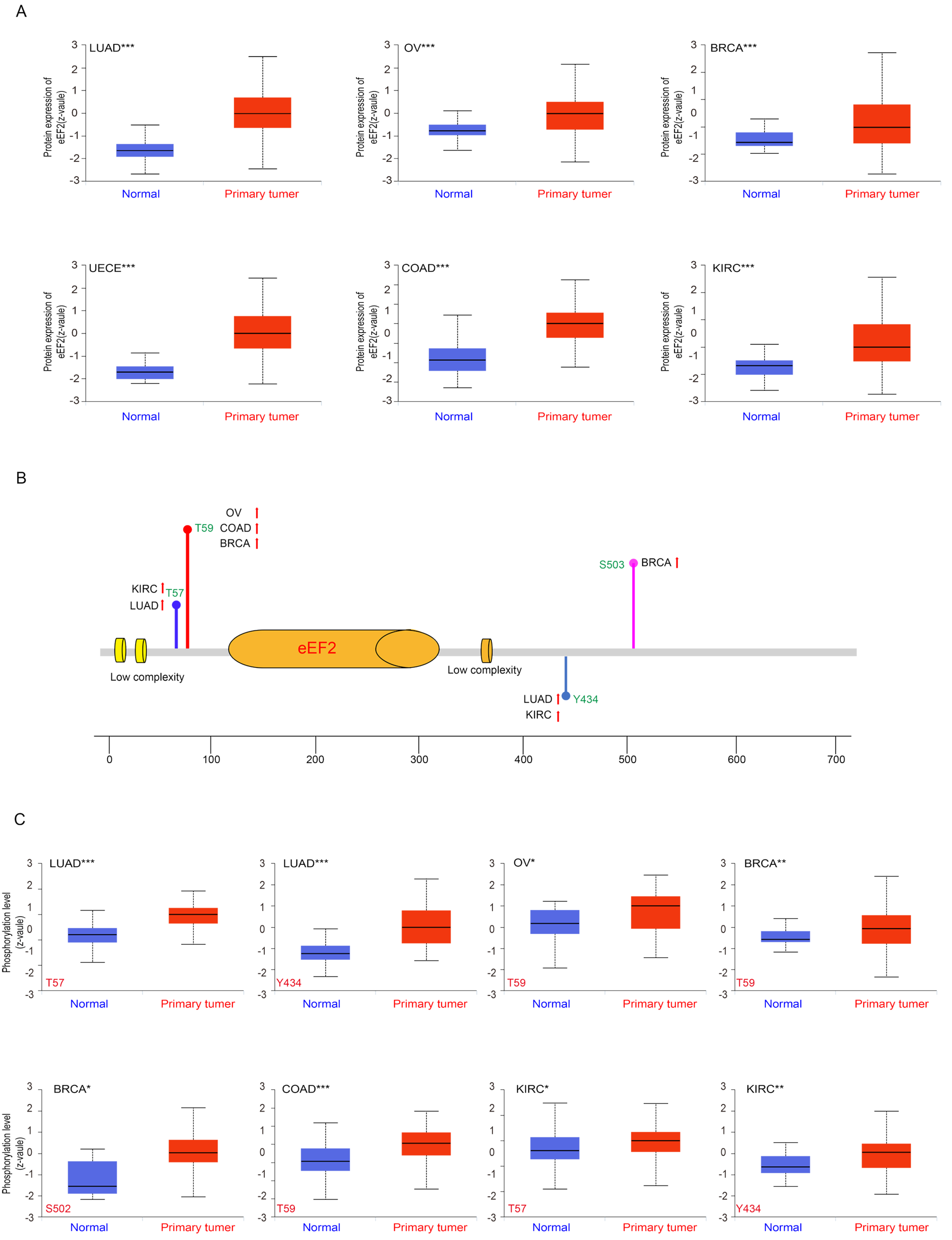

A
LUAD***
OV***
BRCA***
UECE***
COAD***
KIRC***
Protein expression of eEF2(z-vaule)
Normal
Primary tumer
B
OV
T59
COAD
BRCA
KIRC
T57
LUAD
S503
BRCA
eEF2
Low complexity
Low complexity
LUAD
Y434
KIRC
0
100
200
300
400
500
600
700
C
LUAD***
LUAD***
OV*
BRCA**
BRCA*
COAD***
KIRC*
KIRC**
Phosphorylation level (z-vaule)
T57
Y434
T59
T59
S502
T59
T57
Y434
Normal
Primary tumer

Figure S1. Expression levels of eEF2 total protein and phosphorylation at specific sites in various cancer types. (A) Analysis of eEF2 total protein expression levels in LUAD, OV, BRCA, UCEC, COAD, KIRC primary tumor tissues compared to normal tissues. (B) Schematic diagram of eEF2 protein domain showing 5 tumor diseases at each phosphorylation site. (C) Phosphorylation levels of eEF2 at T57, T59, S502, and Y434 sites in COAD, OV, KIRC, LUAD, and BRCA primary tumors and corresponding normal tissues. Statistical significance is indicated: *$p < 0.05$; **$p < 0.01$; ***$p < 0.001$.
